# Supplementary material for: In Vitro Digestion Assessment (Standard vs. Older Adult Model) on Antioxidant Properties and Mineral Bioaccessibility of Fermented Dried Lentils and Quinoa
Source: Molecules. 2023 Oct 27;28(21):7298. doi: 10.3390/molecules28217298 (PMC10649959; doi:10.3390/molecules28217298)
Supplement: Supplementary file 1 [file molecules-28-07298-s001.zip › molecules-2635991-supplementary.pdf]

ELECTRONIC SUPPLEMENTARY MATERIAL

In vitro digestion assessment (standard vs. older adult model) on antioxidant properties and mineral bioaccessibility of fermented-dried lentils and quinoa

Janaina Sánchez-García[1], Sara Muñoz-Pina[1*], Jorge García-Hernández[2], Amparo Tárrega[3], Ana Heredia[1], Ana Andrés[1]

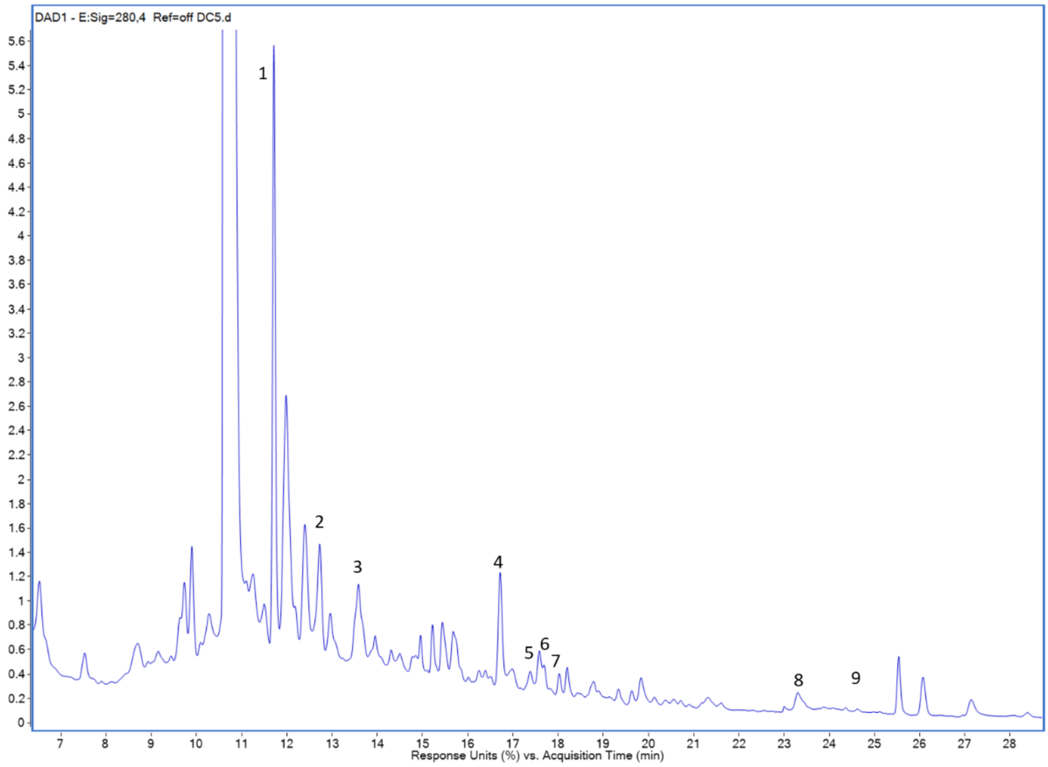

Figure S1. Chromatographic profile of Unfermented Pardina Lentil (UFPL) digested (standard) extracted at 280 nm. 1: 4-O-Caffeoylquinic; 2: 4-Hydroxybenzoic acid; 3: Vanillic acid; 4: p-Coumaric acid; 5: Ferulic acid; 6: Quercitrin; 7: Apigenin-7-glucoside; 8: Quercetin; 9: trans-Cinnamic acid. Note: not all the compounds are quantified at 280 nm.

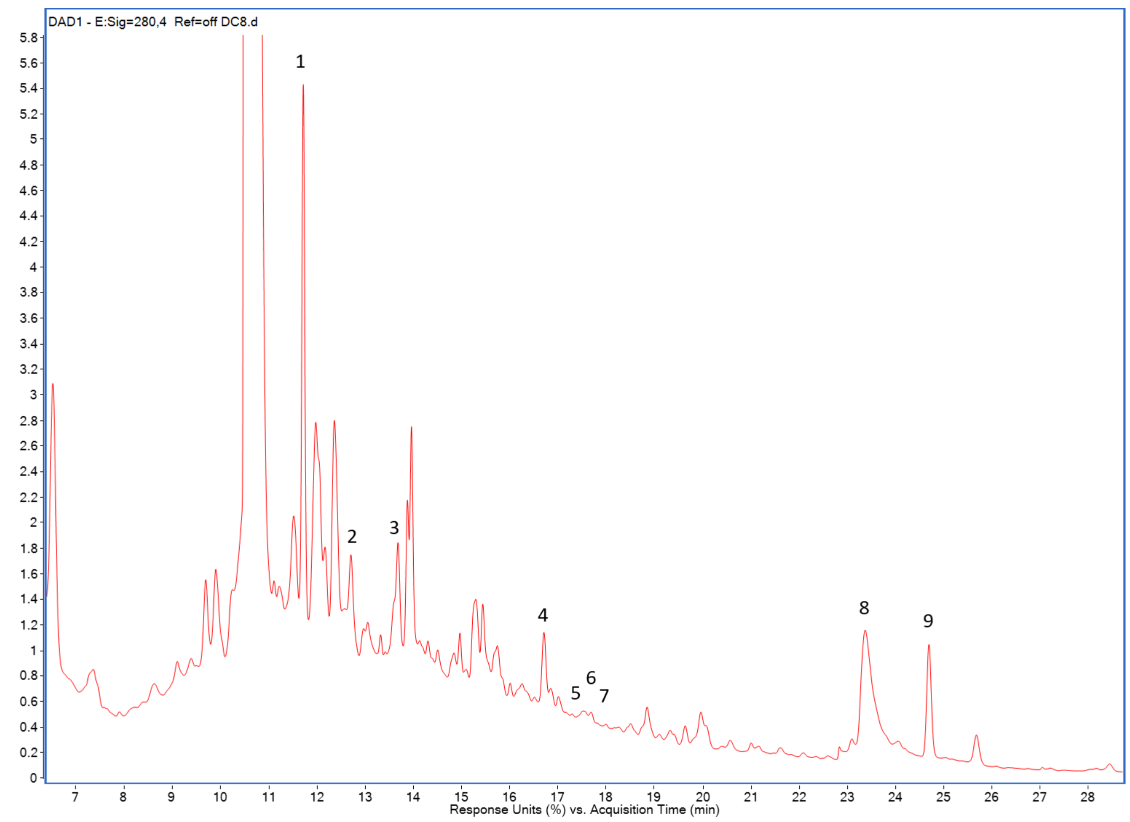

Figure S2. Chromatographic profile of Fermented Pardina Lentil (FPL) digested (standard) extracted at 280 nm. 1: 4-O-Caffeoylquinic; 2: 4-Hydroxybenzoic acid; 3: Vanillic acid; 4: p-Coumaric acid; 5: Ferulic acid; 6: Quercitrin; 7: Apigenin-7-glucoside; 8: Quercetin; 9: trans-Cinnamic acid. Note: not all the compounds are quantified at 280 nm.

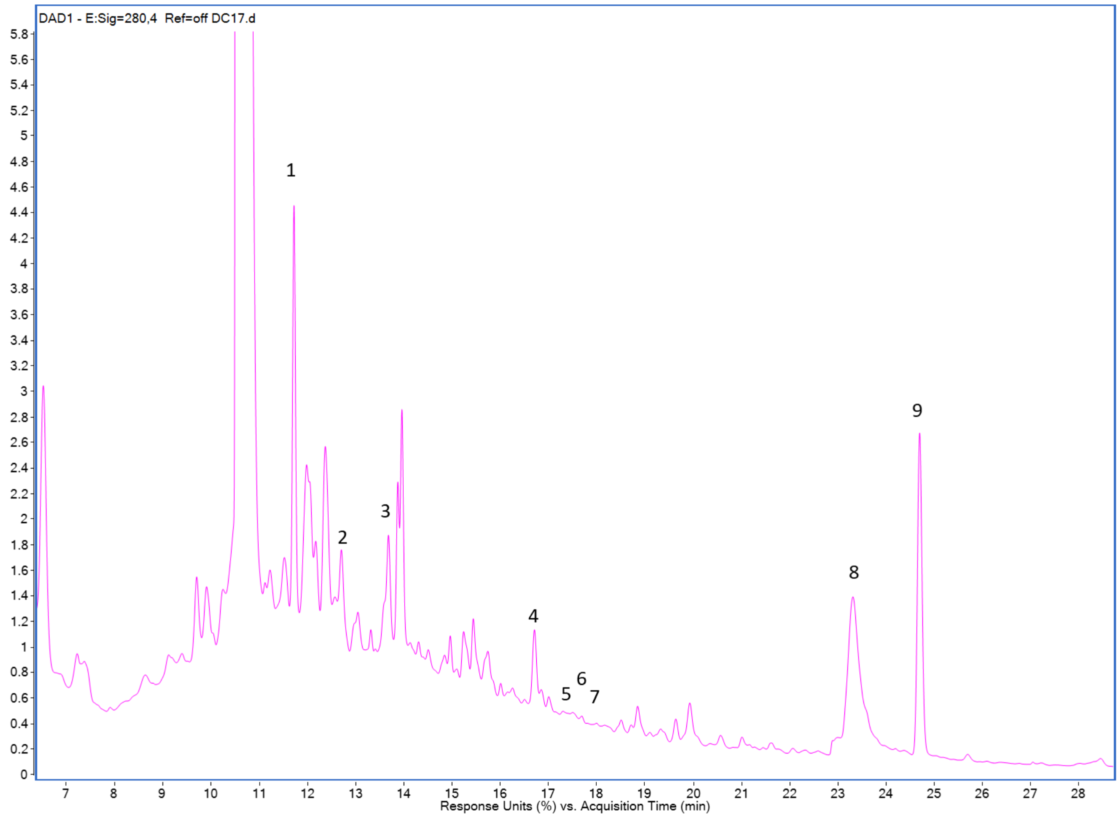

Figure S3. Chromatographic profile of Fermented-dried at 70 °C Pardina Lentil (FPL-70) digested (standard) extracted at 280 nm. 1: 4-O-Caffeoylquinic; 2: 4-Hydroxybenzoic acid; 3: Vanillic acid; 4: p-Coumaric acid; 5: Ferulic acid; 6: Quercitrin; 7: Apigenin-7-glucoside; 8: Quercetin; 9: trans-Cinnamic acid. Note: not all the compounds are quantified at 280 nm.

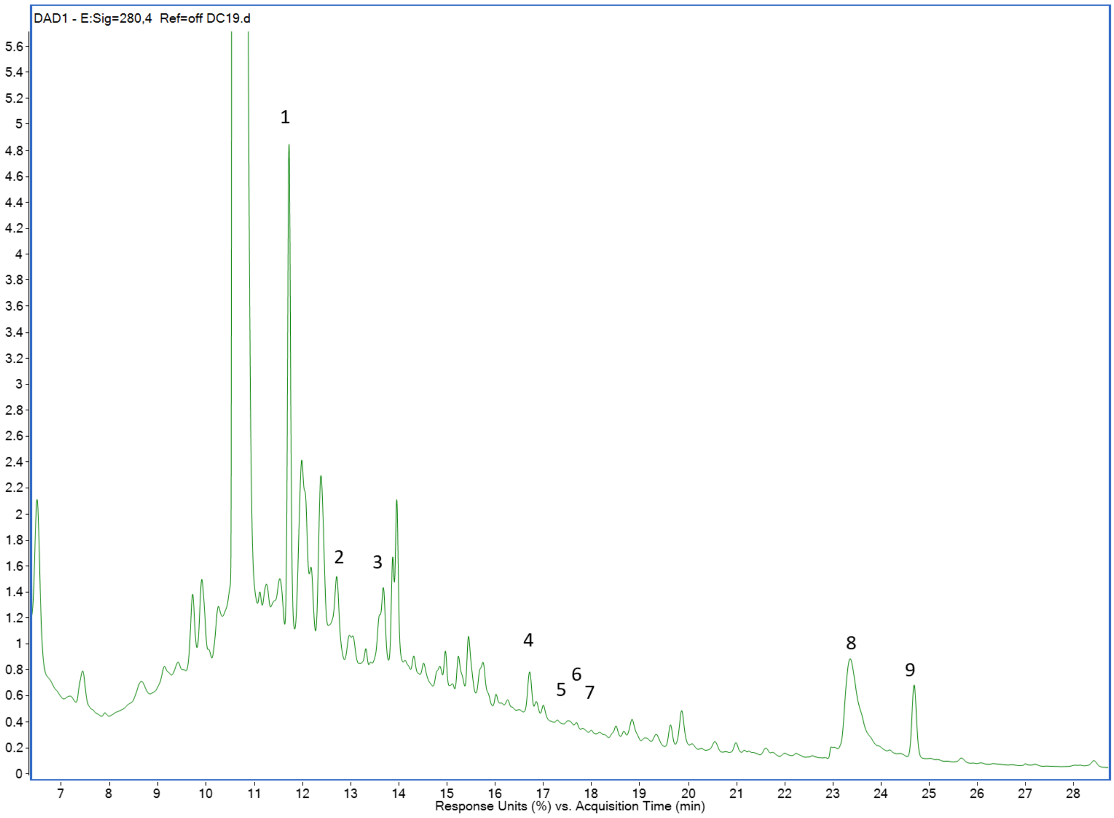

Figure S4. Chromatographic profile of Fermented-lyophilised Pardina Lentil (FPL-L) digested (standard) extracted at 280 nm. 1: 4-O-Caffeoylquinic; 2: 4-Hydroxybenzoic acid; 3: Vanillic acid; 4: p-Coumaric acid; 5: Ferulic acid; 6: Quercitrin; 7: Apigenin-7-glucoside; 8: Quercetin; 9: trans-Cinnamic acid. Note: not all the compounds are quantified at 280 nm.

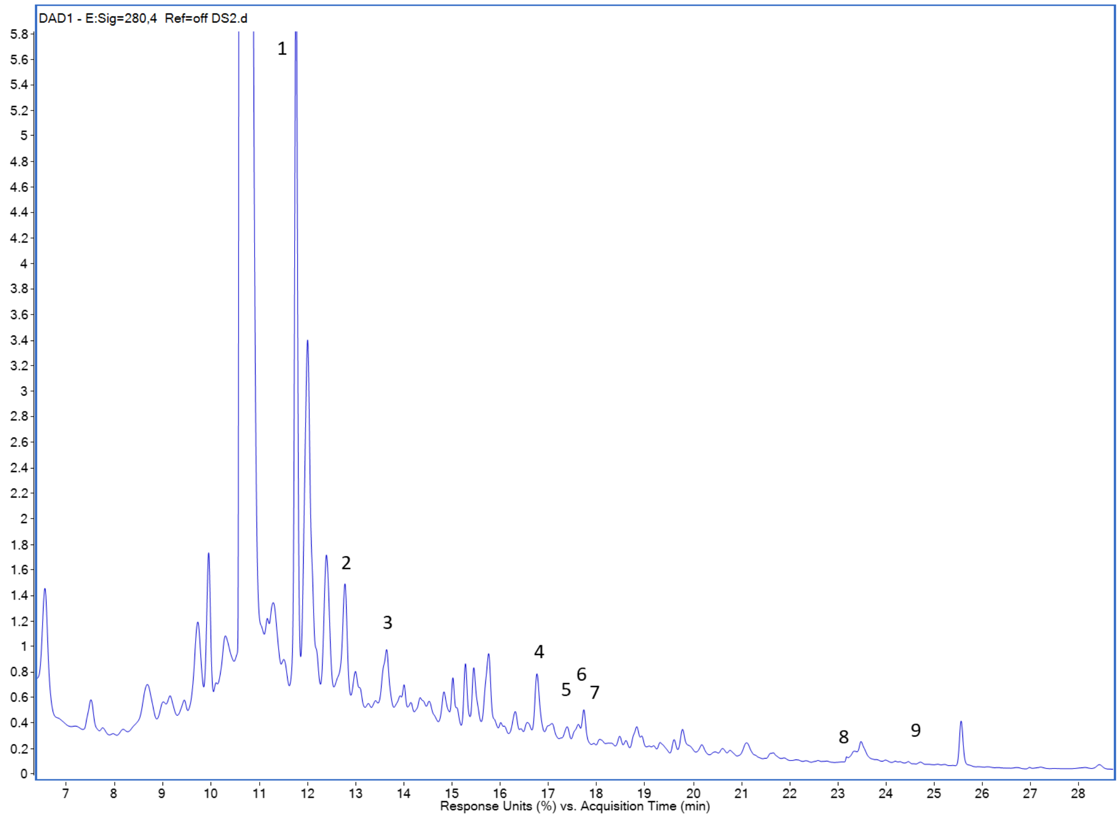

Figure S5. Chromatographic profile of Unfermented Pardina Lentil (UFPL) digested (Older adult) extracted at 280 nm. 1: 4-O-Caffeoylquinic; 2: 4-Hydroxybenzoic acid; 3: Vanillic acid; 4: p-Coumaric acid; 5: Ferulic acid; 6: Quercitrin; 7: Apigenin-7-glucoside; 8: Quercetin; 9: trans-Cinnamic acid. Note: not all the compounds are quantified at 280 nm.

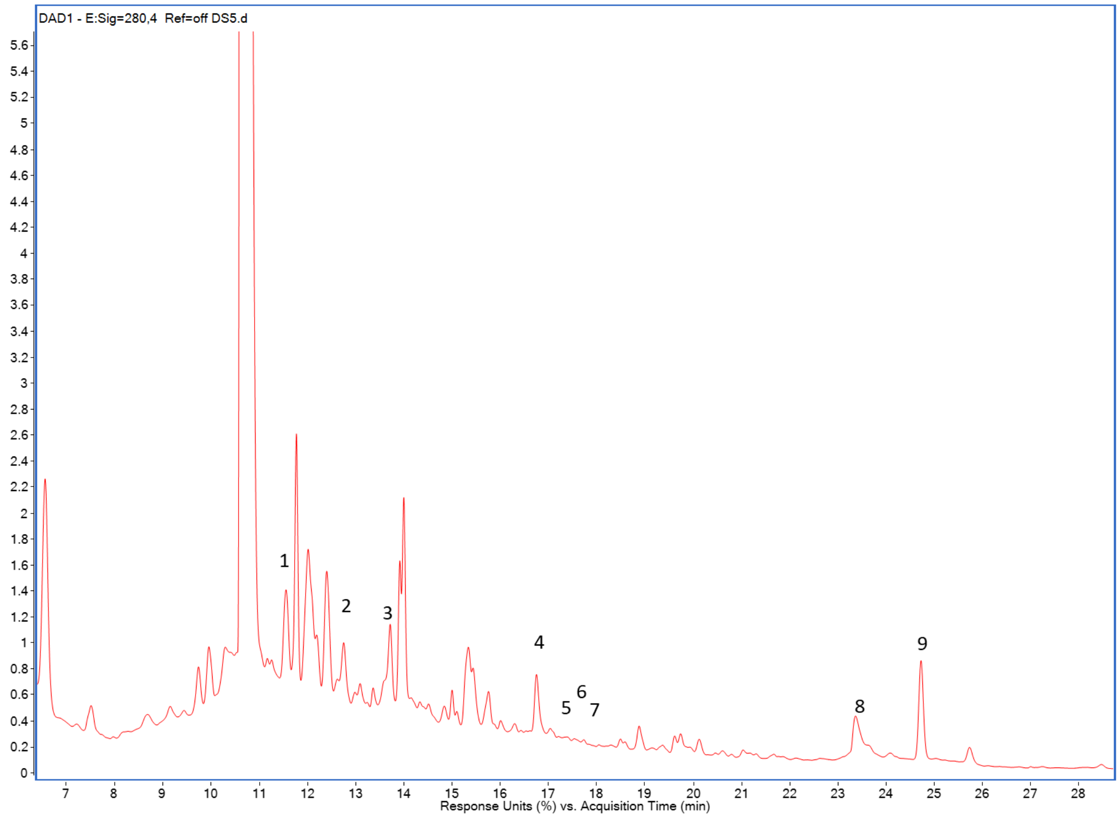

Figure S6. Chromatographic profile of Fermented Pardina Lentil (FPL) digested (Older adult) extracted at 280 nm. 1: 4-O-Caffeoylquinic; 2: 4-Hydroxybenzoic acid; 3: Vanillic acid; 4: p-Coumaric acid; 5: Ferulic acid; 6: Quercitrin; 7: Apigenin-7-glucoside; 8: Quercetin; 9: trans-Cinnamic acid. Note: not all the compounds are quantified at 280 nm.

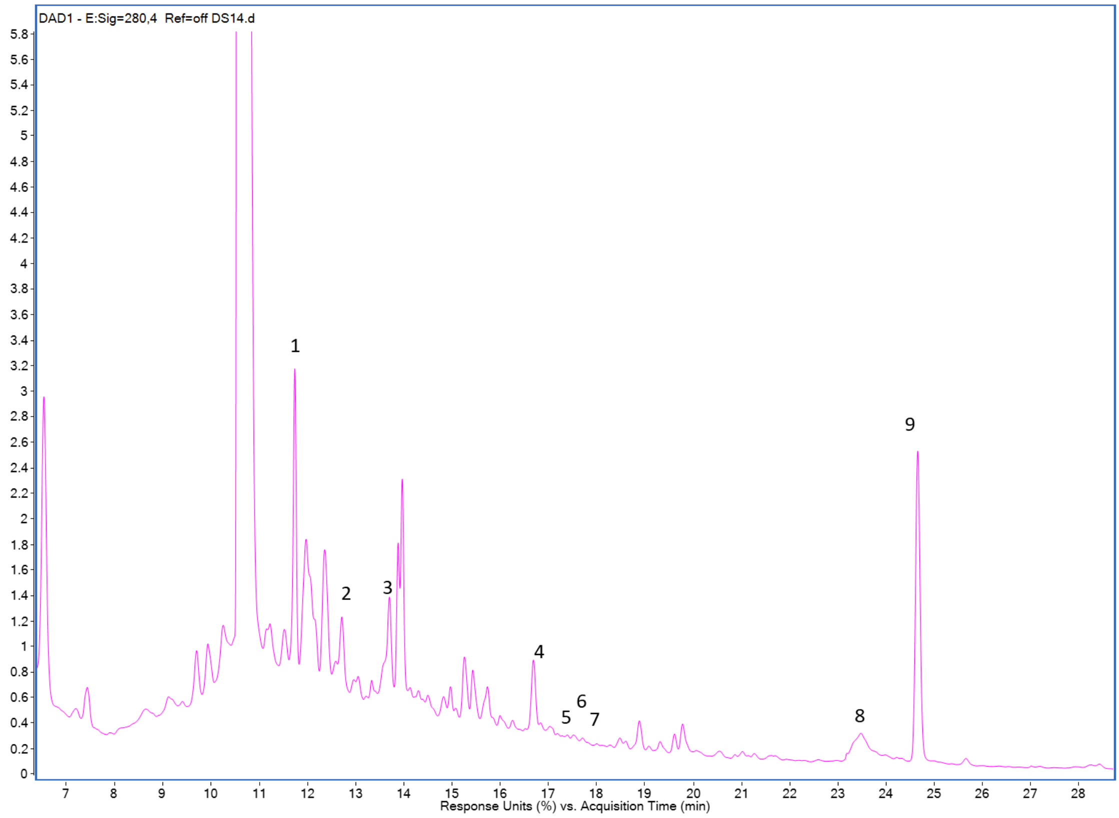

Figure S7. Chromatographic profile of Fermented-dried at 70 °C Pardina lentil (FPL-70) digested (Older adult) extracted at 280 nm. 1: 4-O-Caffeoylquinic; 2: 4-Hydroxybenzoic acid; 3: Vanillic acid; 4: p-Coumaric acid; 5: Ferulic acid; 6: Quercitrin; 7: Apigenin-7-glucoside; 8: Quercetin; 9: trans-Cinnamic acid. Note: not all the compounds are quantified at 280 nm.

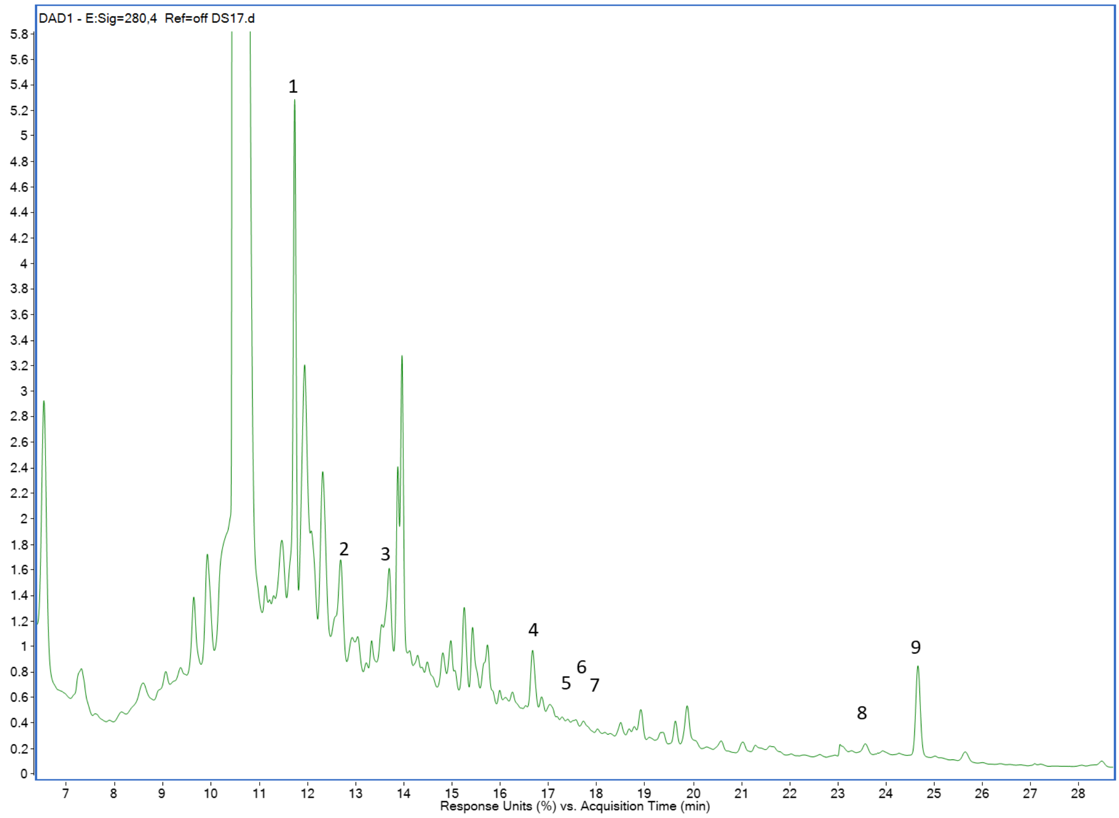

Figure S8. Chromatographic profile of Fermented-lyophilised Pardina Lentil (FPL-L) digested (Older adult) extracted at 280 nm. 1: 4-O-Caffeoylquinic; 2: 4-Hydroxybenzoic acid; 3: Vanillic acid; 4: p-Coumaric acid; 5: Ferulic acid; 6: Quercitrin; 7: Apigenin-7-glucoside; 8: Quercetin; 9: trans-Cinnamic acid. Note: not all the compounds are quantified at 280 nm.
